# Association between concomitant csDMARDs and clinical response to TNF inhibitors in overweight patients with axial spondyloarthritis

**DOI:** 10.1186/s13075-019-1849-3

**Published:** 2019-02-20

**Authors:** Borja Hernández-Breijo, Chamaida Plasencia-Rodríguez, Victoria Navarro-Compán, Ana Martínez-Feito, Andrea Jochems, Eva L. Kneepkens, Gerrit J. Wolbink, Theo Rispens, Cristina Diego, Dora Pascual-Salcedo, Alejandro Balsa

**Affiliations:** 1 Immuno-Rheumatology Research Group, IdiPaz, University Hospital La Paz, Paseo de La Castellana 261, 28046 Madrid, Spain; 2 Immunology, University Hospital La Paz, Madrid, Spain; 3 Rheumatology, University Hospital La Paz, Madrid, Spain; 4 Rheumatology, READE, Amsterdam, Netherlands; 5 Immunopathology, Sanquin, Amsterdam, Netherlands

**Keywords:** Axial spondyloarthritis, TNF inhibitors, Concomitant csDMARDs, Body mass index, Clinical response

## Abstract

**Background:**

The aim of our study was to investigate the influence of conventional synthetic disease-modifying anti-rheumatic drugs (csDMARDs) and body mass index (BMI) on circulating drug levels and clinical response to tumour necrosis factor inhibitor (TNFi) therapy in axial spondyloarthritis (axSpA) patients.

**Methods:**

Prospective observational study during 1 year with 2 cohorts (Madrid and Amsterdam) including 180 axSpA patients treated with standard doses of infliximab or adalimumab. Patients were stratified by BMI, being 78 (43%) normal weight (18.5–24.9 kg/m^2^) and 102 (57%) overweight/obese (≥ 25.0 kg/m^2^). After the first year of treatment, TNFi trough levels were measured by capture ELISA. Clinical response to TNFi was defined as ∆BASDAI ≥ 2 and clinical remission as BASDAI < 2 and CRP ≤ 5 mg/L. Logistic regression models were employed to analyse the association between concomitant csDMARDs and BMI with drug levels and clinical response.

**Results:**

Seventy-nine patients (44%) received concomitant csDMARDs. The administration of concomitant csDMARDs (OR 3.82; 95% CI 1.06–13.84) and being normal weight (OR 18.38; 95% CI 2.24–150.63) were independently associated with serum TNFi drug persistence. Additionally, the use of concomitant csDMARDs contributed positively to achieve clinical response (OR 7.86; 95% CI 2.39–25.78) and remission (OR 4.84; 95% CI 1.09–21.36) in overweight/obese patients, but no association was found for normal-weight patients (OR 1.10; 0.33–3.58).

**Conclusions:**

The use of concomitant csDMARDs with TNFi may increase the probability of achieving clinical response in overweight/obese axSpA patients. Further research studies including larger cohorts of patients need to be done to confirm it.

**Electronic supplementary material:**

The online version of this article (10.1186/s13075-019-1849-3) contains supplementary material, which is available to authorized users.

## Introduction

In patients suffering from spondyloarthritis (SpA), conventional synthetic disease-modifying anti-rheumatic drugs (csDMARDs), such as methotrexate (MTX) and sulfasalazine (SSZ), can be useful for treating peripheral joint manifestations. However, they have not shown to be efficient for axial manifestations and therefore are not recommended by ASAS-EULAR to control axial disease [1].

Nevertheless, it has been described that the phenomenon of immunogenicity, an immune response that may hamper the efficacy of TNF inhibitor (TNFi) monoclonal antibodies [2, 3], can be modulated by co-administration of csDMARDs. To this regard, concomitant use of certain csDMARDs, especially MTX, may ameliorate the immunogenicity of infliximab and adalimumab by reducing anti-drug antibody (ADA) formation and therefore maintaining serum drug circulating levels and prolonging drug survival [4, 5].

However, immunogenicity is not the only cause for losing response to TNFi therapy. Other factors may be of influence. Currently, obesity is being considered as a risk factor for worsening clinical parameters of disease activity in patients with ankylosing spondylitis (AS). The increase of pro-inflammatory adipokines during obesity, such as vinfastin, correlates with the progression of radiographic damage in AS [6]. Furthermore, an increased body mass index (BMI) has been recently found to be associated with significantly lower response rates to TNFi in patients with axial (axSpA) [7, 8]. Some studies have furthermore demonstrated that high BMI is negatively associated with TNFi serum drug levels [9, 10].

In summary, both BMI and csDMARDs are associated with serum TNFi level persistence, but only BMI seems to be associated on clinical response to this therapy. To extent the knowledge on the relationship of these factors, the aim of this study was to investigate the influence of both BMI and concomitant csDMARDs on serum TNFi persistence as well as on the clinical response in patients with axSpA [7, 8].

## Methods

### Study design and patients

This was a prospective observational study. Data from two different cohorts including patients diagnosed of axSpA treated with standard doses of infliximab or adalimumab (infliximab 5 mg/kg at 0, 2, 6 weeks and later every 8 weeks; adalimumab 40 mg/2 weeks) according to the clinical practice guidelines were analysed. No modifications in the dose or interval of administration were carried out. Patients were included from June 2000 to June 2015. All included patients were diagnosed as axSpA according to physician criteria. The adherence to ASAS criteria was verified before including them into the study [11, 12]. When the datasets were locked (November 2016), a total of 246 patients had been enrolled in these cohorts: 166 from a Spanish inception cohort at the Department of Rheumatology of La Paz University Hospital (Madrid) and 80 from a Netherland cohort at the Department of Rheumatology of Reade Center (Amsterdam). For this study, only patients who had baseline BMI data available and serum drug levels measured or disease activity assessment during the first year of TNFi therapy after 1 year of starting infliximab or adalimumab were selected (*n* = 180). Out of these, 81% were naïve to biological therapy. It was described in a flowchart in Additional file 1. Non-radiographic axSpA and radiographic axSpA were defined according to physician and clinical practice, but imaging was not systematically read for this study. The study was conducted according to the guidelines of the 1975 Declaration of Helsinki. Approval was obtained from the Institutional Ethics Committee from both centres.

### Clinical disease activity assessment

Disease activity was assessed by *Bath Ankylosing Spondylitis Disease Activity Index* (BASDAI) and *Ankylosing Spondylitis Disease Activity Score* (ASDAS) at baseline (before starting TNFi treatment) and after 1 year of treatment. Clinical response to TNFi was defined as ΔBASDAI ≥ 2 and as ΔASDAS ≥ 1.1. Clinical remission was defined as BASDAI < 2 and CRP ≤ 5 mg/L and as ASDAS < 1.3. For all analyses, BASDAI was considered as the main variable due to the later development of the ASDAS in 2010, which explained why fewer patients from both cohorts were evaluated by this score (*n* = 170 patients with BASDAI score and *n* = 141 patients with ASDAS).

### Determination of drug levels and anti-drug antibodies

Blood samples were collected at 24 h maximum before drug administration for adalimumab or immediately before intravenous infusions for infliximab.

Serum drug concentrations were determined by a capture enzyme-linked immunosorbent assay (ELISA), as described previously [13], using specific biotin-conjugated anti-idiotype antibodies to infliximab and adalimumab. Threshold values for positive drug levels were 10 ng/mL for infliximab and 5 ng/mL for adalimumab.

Serum anti-drug antibody (ADA) levels were analysed by radioimmunoassay (RIA), as described previously [14], using ^125^I-infliximab F(ab’)_2_ or ^125^I-adalimumab F(ab’)_2_. Threshold values for positive ADA levels were 12 AU/mL.

### Statistical analyses

First, descriptive analysis was employed. According to BMI, patients were classified as normal weight (18.5–24.9 kg/m^2^) and overweight/obese (≥ 25.0 kg/m^2^). Differences between the groups were assessed using the unpaired *t* test or Mann-Whitney *U* test for continuous variables depending on the distribution and Fisher’s exact test for ordinal variables. Second, the association between concomitant csDMARDs and BMI with drug persistence and clinical response to TNFi at 1 year was analysed through univariate and multivariate logistic regression models. Interactions with age, gender, HLA-B27, BMI, csDMARDs and symptom duration were tested. If relevant interactions were found, analyses were stratified. If not, variables were entered as covariates. Additionally, the type of TNFi for drug persistence outcome and baseline disease activity (BASDAI and CRP) for clinical response outcome were included as covariates. Results are shown as odds ratio (OR) and 95% confidence interval (CI).

For TNFi levels, the last observation carried forward method (LOCF) was performed to include patients who dropped out before 1 year of follow-up (*n* = 43). Statistical significance was calculated considering *p* value < 0.05 statistically significant. The Statistical Package for the Social Sciences version 24 (SPSS, Chicago, IL, USA) was used for the analyses.

## Results

### Baseline characteristics

From a total of 246 patients, 180 patients with axSpA starting infliximab (*n* = 74) or adalimumab (*n* = 106) were included in the study. Patient and disease characteristics at baseline are shown in Table 1. There were no differences between the total group of patients and the patients included in the study. A total of 79 (44%) patients received concomitant csDMARD distributed as follows: MTX (25; 14%), SSZ (36; 20%) and MTX+SSZ (18; 10%). According to BMI, 78 (43%) patients were normal weight (18.5–24.9 kg/m^2^) and 102 (57%) were overweight/obese (≥ 25.0 kg/m^2^). Overall, no differences between both groups of BMI were found, except for age, sex, disease duration, TNFi type and concomitant sulfasalazine.

**Table 1:** Baseline characteristics of patients included in the study

Characteristics	Total patients (*n* = 246)	Included patients (*n* = 180)	Included patients BMI ≤ 25 (*n* = 78)	Included patients BMI > 25 (*n* = 102)
Age (years)	47.6 ± 13.1	47.0 ± 12.7	42.9 ± 11.7	50.1 ± 12.6***
Male	146 (59)	107 (59)	39 (50)	68 (67)*
Disease duration (years)	10 (5–16)	8 (5–16)	7 (3–13)	9 (6–18)**
SpA subtype				
Ankylosing spondylitis	179 (78)	140 (78)	64 (82)	76 (74)
Non-radiographic SpA	49 (22)	40 (22)	14 (18)	26 (26)
HLA-B27	174 (74)	131 (73)	61 (78)	70 (69)
Psoriasis/IBD/uveitis	71 (29)	58 (32)	27 (35)	31 (30)
Peripheral arthritis	97 (40)	86 (48)	38 (49)	48 (47)
BASDAI	5.8 ± 2.0	5.8 ± 2.0	5.5 ± 2.1	6.0 ± 1.9
ASDAS	3.3 ± 0.9	3.3 ± 1.0	3.2 ± 1.0	3.4 ± 1.0
ESR (mmHg)	17 (7–33)	17 (7–31)	19 (6–34)	16 (7–31)
CRP (mg/L)	5 (2–15)	6 (3–17)	7 (2–20)	5 (3–15)
Previous TNFi	43 (18)	35 (19)	13 (17)	22 (21)
TNFi type				
Infliximab	107 (43)	74 (41)	25 (32)	49 (48)*
Adalimumab	139 (57)	106 (59)	53 (68)	53 (52)*
csDMARD				
Any csDMARD	102 (42)	79 (44)	31 (40)	48 (47)
MTX	34 (15)	25 (14)	12 (15)	13 (13)
SSZ	46 (21)	36 (20)	10 (13)	26 (25)*
MTX and SSZ	22 (9)	18 (10)	9 (11)	9 (9)
Prednisone	22 (9)	20 (11)	11 (14)	9 (9)

The table shows mean ± SD, median (IQR) or absolute number (percentage) for all patients included (*n* = 180). The results are also stratified by body mass index (BMI). *p* value < 0.05 was considered statistically significant. Significant statistical differences between the groups of included patients, stratified by BMI: *p < 0.05, **p < 0.01, ***p < 0.001. *SpA* spondyloarthritis, *HLA-B27* human leucocyte antigen B27, *ESR* erythrocyte sedimentation rate, *CRP* C-reactive protein, *IBD* inflammatory bowel disease, *BASDAI* Bath Ankylosing Spondylitis Disease Activity Index, *ASDAS* Ankylosing Spondylitis Disease Activity Index, *TNFi* tumour necrosis factor inhibitors, *csDMARD* conventional synthetic disease-modifying anti-rheumatic drug, *MTX* methotrexate, *SSZ* sulfasalazine

### Effect of concomitant csDMARDs and BMI on persistence of TNFi in serum

A total of 157 patients (87%) had detectable circulating TNFi levels after 1 year of treatment. For this outcome, no significant interaction with other variables was found. Univariable analyses were performed to analyse the association between the persistence of serum TNFi and each variable included in Table 1. A significant association was found for being male (OR 0.32; 95% CI 0.11–0.89), disease duration (OR 0.94; 95% CI 0.90–0.98), being normal weight (OR 9.85; 95% CI 2.23–43.44) and concomitant csDMARDs (OR 2.71; 95% CI 1.03–7.14). In the multivariable logistic regression model, disease duration (OR 0.93; 95% CI 0.88–0.99), concomitant csDMARDs (OR 3.82; 95% CI 1.06–13.84) and especially being normal weight (OR 18.38; 95% CI 2.24–150.63) remained independently associated with serum TNFi persistence after 1 year of treatment (Table 2). Specifically, all the patients concomitantly treated with MTX [± SSZ] showed detectable TNFi levels after 1 year of treatment. At the same time, lower percentages of patients showing detectable TNFi levels after 1 year of treatment were found in the groups under TNFi monotherapy (83/101; 82%) or concomitantly treated with SSZ alone (31/36; 86%).

**Table 2:** Association between csDMARDs and BMI with the presence of serum TNFi after 1 year of treatment

	OR	95% CI
Age	1.04	0.98–1.11
Male	0.59	0.17–2.14
Disease duration	0.93	0.88–0.99*
TNFi type (ref: infliximab)	0.46	0.15–1.39
csDMARD (ref: use of any csDMARD)	3.82	1.06–13.84*
BMI (ref: normal weight)	18.38	2.24–150.63*

The adjusted multivariable logistic regression analysis included 161 patients. Odds ratio (OR) and 95% confidence interval (CI) were calculated. *p value < 0.05 was considered statistically significant. *TNFi* tumour necrosis factor inhibitors, *csDMARD* conventional synthetic disease-modifying anti-rheumatic drug, *BMI* body mass index

These results were similar, regardless of the TNFi type. Patients receiving TNFi as monotherapy showed a higher percentage of undetectable drug levels than patients treated with MTX [± SSZ] after 1 year of treatment, regardless of the TNFi type (28% for infliximab, 17% for adalimumab; *p* = 0.2).

Concerning the association with BMI, patients who showed undetectable TNFi presented mainly overweight/obese (*n* = 21/23; 91%), regardless of the TNFi type.

### Effect of concomitant csDMARDs and BMI on ADA development

Thirty-eight out of 180 patients (21%) developed ADA at 1 year of TNFi treatment. The development of ADA for each therapeutic group was as follows: 30/101 (30%) patients under TNFi monotherapy, 7/36 (19%) patients treated with SSZ (*p* = 1.0) and 1/43 (2%) patients treated with MTX [± SSZ] (*p* = 0.03). The provided *p* values were obtained by comparison with TNFi monotherapy. ADA development was significantly negatively associated with TNFi persistence (*β* − 3.94; OR 0.02; 95% CI 0.005–0.72).

Moreover, a higher BMI was associated with the development of ADA (*p* = 0.002). Forty-five percent of obese patients (15/35) were ADA positive at 1 year of treatment. On the other hand, ADA development was lower in overweight (11/69; 16%) as well as normal-weight (12/78; 15%) patients. No statistical differences were observed between the overweight and normal-weight groups.

### Effect of concomitant csDMARDs and BMI on clinical outcomes

#### Effect on clinical response

Patients had active disease at baseline, as indicated by a mean BASDAI of 5.8 and a mean ASDAS of 3.3. After 1 year of TNFi treatment, 48% (*n* = 68) of the patients achieved BASDAI clinical response. For this outcome, a relevant interaction between csDMARDs and BMI with clinical response was found (Wald chi-square value 3.63; *p* = 0.057), and therefore, the results were stratified according to BMI. The results showed that the use of concomitant csDMARDs contributed positively to achieve BASDAI clinical response in overweight/obese patients (OR 7.86; 95% CI 2.39–25.78); however, no association was found for normal-weight patients (OR 1.10; 95% CI 0.33–3.58) (Table 3).

**Table 3:** Association between csDMARDs and clinical response (∆BASDAI ≥ 2.0) or remission (BASDAI < 2 and CRP ≤ 5 mg/L) at 1 year, stratified for body mass index

	BMI ≤ 25 (*n* = 60; 42%)		BMI > 25 (*n* = 81; 58%)	
	OR	95% CI	OR	95% CI
Clinical response				
Any csDMARD	1.10	0.33–3.58	7.86	2.39–25.78*
MTX [± SSZ]	1.04	0.25–4.25	9.82	2.13–45.20*
SSZ	1.18	0.25–5.63	6.86	1.85–25.40*
Remission				
Any csDMARD	0.76	0.20–2.86	4.84	1.09–21.36*
MTX [± SSZ]	0.60	0.11–3.18	5.56	0.84–36.52
SSZ	0.99	0.17–5.64	4.35	0.77–24.54

The adjusted multivariable logistic regression analysis included 141 patients. Two different models are presented for the following outcomes: clinical response and remission. Odds ratio (OR) and 95% confidence interval (CI) were calculated. All models were adjusted for age, gender, disease duration, HLA-B27, baseline BASDAI and CRP. *p value < 0.05 was considered statistically significant. *BMI* body mass index, *HLA-B27* human leucocyte antigen B27, *CRP* C-reactive protein, *BASDAI* Bath Ankylosing Spondylitis Disease Activity Index, *csDMARD* conventional synthetic disease-modifying anti-rheumatic drug, *MTX* methotrexate, *SSZ* sulfasalazine

Furthermore, the influence of csDMARD type on these associations was also investigated. Concomitant MTX [± SSZ] (OR 9.82; 95% CI 2.13–45.20) as well as concomitant SSZ alone (OR 6.86; 95% CI 1.85–25.40) contributed to achieve BASDAI clinical response in overweight/obese patients.

#### Effect on clinical remission

After 1 year of treatment with TNFi, 25% (*n* = 35) of patients achieved clinical remission. Similarly to the results observed for clinical response, the analysis revealed that the use of csDMARDs contributes to achieve BASDAI clinical remission in overweight/obese patients (OR 4.84; 95% CI 1.09–21.36) (Table 3). The individual results for csDMARD type were analysed, but neither concomitant MTX [± SSZ] (OR 5.56; 95% CI 0.84–36.52) nor concomitant SSZ (OR 4.35; 0.77–24.54) showed significant results.

#### Sensitivity analyses

Finally, sensitivity analyses were performed employing ASDAS definition for clinical response using data from 119 patients in which ASDAS was available. Out of these, 45% (*n* = 53) of patients achieved clinical response and 29% (*n* = 34) remission. The results found were consistent with the evaluation with BASDAI, being the probability of achieving clinical response in overweight/obese patients increased by the use of concomitant csDMARD (Additional file 1). However, these results were not statistically significant, due to the smaller sample size.

## Discussion

This study investigates the influence of concomitant csDMARDs and BMI on circulating drug levels as well as on the clinical response to TNFi therapy in axSpA patients. The observed results show that both concomitant csDMARDs and especially BMI were associated with TNFi drug persistence. Furthermore, the concomitant treatment with csDMARDs (MTX and/or SSZ) was also associated with good clinical response in overweight/obese axSpA patients receiving TNFi but not in normal-weight patients.

According to previous data, BMI and csDMARDs were found to have an influence on TNFi clearance [8]. Particularly, MTX was more effective in reducing immunogenicity. However, the effect of BMI seems to be more relevant. Different studies have suggested that higher BMI is associated with a higher volume of distribution in the central compartment and therefore with increased TNFi clearance [7–10].

Up to date, there are no clear data about the clinical superiority of co-therapy csDMARDs+TNFi vs. TNFi in monotherapy for the treatment of axSpA [1]. Recently, Sepriano et al. indicated that co-medication with csDMARDs do not prolong TNFi retention in axSpA [15]. To this regard, we observed that the use of concomitant csDMARDs have no influence on the clinical response for normal-weight patients and therefore support previous data. However, csDMARDs were associated with a higher probability of having both BASDAI clinical response and clinical remission in patients being overweight or obese. An explanation for this association could be the fact that obesity might be considered as additional inflammation where pro-inflammatory adipokines, such as TNFα, IL1β, IL6 or MCP1, are segregated by adipose tissue and macrophages [16]. Therefore, the concomitant treatment with immunomodulators (csDMARDs) would allow decreasing the production of these adipokines helping to restore inflammation balance [17, 18]. TNFi would achieve the clinical outcome, once decreased the effect of the overweight/obese pro-inflammatory environment.

Nevertheless, the results of this study need to be interpreted with caution. It is well known that studies testing treatment effects without blinded and random treatment allocation are at risk for confounding by indication. In our study, csDMARD prescription was not randomised and was done according to the clinical criteria by the treating physician. Patients who were prescribed csDMARDs may be inherently different from those who did not take these drugs, and such differences can drive the positive association rather than the treatment itself. So, confounding by indication needs to be considered as the main limitation of this study. On the other hand, we are convinced that the reason to prescribe concomitant csDMARDs were not based on BMI because this is not the clinical practice in the participant centres.

This study presents other limitations that need to be kept in mind when interpreting the results: (i) Statistical analyses could not be adjusted for smoking habit because it was not registered. (ii) Some patients were included before 2010, when ASDAS was not available, and therefore, the sample size for these analyses was reduced; the results obtained for this analysis showed a similar trend to that of BASDAI clinical response but were not statistically significant. The lower number of patients with ASDAS registered may be an explanation. Furthermore, a different explanation could be that ASDAS was not really influenced by BMI. In this regard, it has recently been published that it would not be necessary to take BMI into account when assessing disease activity by ASDAS in axSpA patients with high BMI [19]. (iii) LOCF was employed for patients who dropped out before 1 year of follow-up (*n* = 43). (iv) csDMARD prescription was not randomised, although prescription followed clinical criteria. (v) Patients are included from two different centres with some differences in the therapeutic strategy. (vi) BMI and csDMARD information was collected only at baseline, and therefore, the possible change on these over the study period was not taken into account. (vii) The compliance in adalimumab, SSZ and MTX administrations could not be ensured in all patients.

## Conclusions

In conclusion, our study suggests that the use of concomitant csDMARDs with TNFi would not be justified in axSpA patients with normal weight as it would not increase the probability of achieving clinical response. Moreover, in agreement with previous data, it also suggests that BMI is associated with lower TNFi serum drug persistence. Therefore, obese patients should be encouraged to achieving normal weight. Nevertheless, based on the results of this study, if losing weight is proven impossible, it can be hypothesised that the co-medication with csDMARDs (MTX or SSZ) could be of additional value to increase treatment success in overweight/obese patients. However, before implementing the results of this study, further research studies with better methodological design including larger cohorts of patients need to confirm them.

## Additional file

Additional file 1: (i) Association between csDMARDs and clinical response (∆ASDAS≥1.1) or remission (ASDAS<1.3) at one year, stratified for body mass index; (ii) Flowchart of patients included in the present study. (DOCX 46 kb)

## Abbreviations

ADA: Anti-drug antibodies
ASDAS: Ankylosing Spondylitis Disease Activity Score
axSpA: Axial spondyloarthritis
BASDAI: Bath Ankylosing Spondylitis Disease Activity Index
BMI: Body mass index
CI: Confidence interval
CRP: C-reactive protein
csDMARD: Conventional synthetic disease-modifying anti-rheumatic drug
ELISA: Enzyme-linked immunosorbent assay
ESR: Erythrocyte sedimentation rate
HLA-B27: Human leucocyte antigen B27
IBD: Inflammatory bowel disease
IL1β: Interleukin-1β
IL6: Interleukin-6
LOCF: Last observation carried forward
MCP1: Monocyte chemoattractant protein 1
MTX: Methotrexate
OR: Odds ratio
RIA: Radioimmunoassay
SSZ: Sulfasalazine
TNF: Tumour necrosis factor
TNFi: TNF inhibitor

## References

[1] Van der Heijde D, Ramiro S, Landewé R, Baraliakos X, Van den Bosch F, Sepriano A (2017). 2016 update of the ASAS-EULAR management recommendations for axial spondyloarthritis. Ann Rheum Dis.

[2] Kneepkens EL, Wei JC, Nurmohamed MT, Yeo KJ, Chen CY, van der Horst-Bruinsma IE (2015). Immunogenicity, adalimumab levels and clinical response in ankylosing spondylitis patients during 24 weeks of follow-up. Ann Rheum Dis.

[3] de Vries MK, Wolbink GJ, Stapel SO, de Vrieze H, van Denderen JC, Dijkmans BA (2007). Decreased clinical response to infliximab in ankylosing spondylitis is correlated with anti-infliximab formation. Ann Rheum Dis.

[4] Jani M, Barton A, Warren RB, Griffiths CE, Chinoy H (2014). The role of DMARDs in reducing the immunogenicity of TNF inhibitors in chronic inflammatory diseases. Rheumatology (Oxford).

[5] Plasencia C, Pascual-Salcedo D, Nuño L, Bonilla G, Villalba A, Peiteado D (2012). Influence of immunogenicity on the efficacy of longterm treatment of spondyloarthritis with infliximab. Ann Rheum Dis.

[6] Syrbe U, Callhoff J, Conrad K, Poddubnyy D, Haibel H, Junker S (2015). Serum adipokine levels in patients with ankylosing spondylitis and their relationship to clinical parameters and radiographic spinal progression. Arthritis Rheumatol.

[7] Micheroli R, Hebeisen M, Wildi LM, Exer P, Tamborrini G, Bernhard J (2017). Impact of obesity on the response to tumor necrosis factor inhibitors in axial spondyloarthritis. Arthritis Res Ther.

[8] Rosas J, Llinares-Tello F, Senabre-Gallego JM, Barber-Vallés X, Santos-Soler G, Salas-Heredia E (2017). Obesity decreases clinical efficacy and levels of adalimumab in patients with ankylosing spondylitis. Clin Exp Rheumatol.

[9] Dreesen E, Gils A, Vermeire S (2018). Pharmacokinetic modeling and simulation of biologicals in inflammatory bowel disease: the dawning of a new era for personalized treatment. Curr Drug Targets.

[10] Ordás I, Mould DR, Feagan BG, Sandborn WJ (2012). Anti-TNF monoclonal antibodies in inflammatory bowel disease: pharmacokinetics-based dosing paradigms. Clin Pharmacol Ther.

[11] Rudwaleit M, Landewe R, van der Heijde D, Listing J, Brandt J, Braun J (2009). The development of Assessment of SpondyloArthritis international Society classification criteria for axial spondyloarthritis (part I): classification of paper patients by expert opinion including uncertainty appraisal. Ann Rheum Dis.

[12] Rudwaleit M, van der Heijde D, Landewe R, Listing J, Akkoc N, Brandt J (2009). The development of Assessment of SpondyloArthritis international Society classification criteria for axial spondyloarthritis (part II): validation and final selection. Ann Rheum Dis.

[13] Pascual-Salcedo D, Plasencia C, Ramiro S, Nuño L, Bonilla G, Nagore D (2011). Influence of immunogenicity on the efficacy of long-term treatment with infliximab in rheumatoid arthritis. Rheumatology (Oxford).

[14] Bloem K, van Leeuwen A, Verbeek G, Nurmohamed MT, Wolbink GJ, van der Kleij D (2015). Systematic comparison of drug-tolerant assays for anti-drug antibodies in a cohort of adalimumab-treated rheumatoid arthritis patients. J Immunol Methods.

[15] Sepriano A, Ramiro S, van der Heijde D, Ávila-Ribeiro P, Fonseca R, Borges J (2016). Effect of comedication with conventional synthetic disease-modifying Antirheumatic drugs on retention of tumor necrosis factor inhibitors in patients with spondyloarthritis: a prospective cohort study. Arthritis Rheumatol.

[16] Medina G, Vera-Lastra O, Peralta-Amaro AL, Jiménez-Arellano MP, Saavedra MA, Cruz-Domínguez MP (2018). Metabolic syndrome, autoimmunity and rheumatic diseases. Pharmacol Res.

[17] Toms TE, Panoulas VF, John H, Douglas KM, Kitas GD (2009). Methotrexate therapy associates with reduced prevalence of the metabolic syndrome in rheumatoid arthritis patients over the age of 60- more than just an anti-inflammatory effect? A cross sectional study. Arthritis Res Ther.

[18] DeOliveira CC, Acedo SC, Gotardo EM, Carvalho Pde O, Rocha T, Pedrazzoli J (2012). Effects of methotrexate on inflammatory alterations induced by obesity: an in vivo and in vitro study. Mol Cell Endocrinol.

[19] Rubio Vargas R, van den Berg R, van Lunteren M, Ez-Zaitouni Z, Bakker PA, Dagfinrud H (2016). Does body mass index (BMI) influence the ankylosing spondylitis disease activity score in axial spondyloarthritis?: data from the SPACE cohort. RMD Open.

