# Supplementary material for: Association between concomitant csDMARDs and clinical response to TNF inhibitors in overweight patients with axial spondyloarthritis
Source: Arthritis Res Ther. 2019 Feb 20;21:66. doi: 10.1186/s13075-019-1849-3 (PMC6383284; doi:10.1186/s13075-019-1849-3)
Supplement: Supplementary file 1 — (i) Association between csDMARDs and clinical response (∆ASDAS≥1.1) or remission (ASDAS<1.3) at one year, stratified for body mass index; (ii) Flowchart of patients included in the present study. (DOCX 46 kb) [file 13075_2019_1849_MOESM1_ESM.docx]

***ADDITIONAL FILES***

**Association between concomitant csDMARDs and clinical response to TNF inhibitors in overweight patients with axial spondyloarthritis**

B. Hernández-Breijo, C. Plasencia-Rodríguez, V. Navarro-Compán, A. Martínez-Feito, A. Jochems, E. L. Kneepkens, G. J. Wolbink, T. Rispens, C. Diego, D. Pascual-Salcedo_,_ A. Balsa

**Association between csDMARDs and clinical response (∆ASDAS≥1.1) or remission (ASDAS<1.3) at one year, stratified for body mass index.**

|  | **BMI≤25**  **(n=53; 44%)** | | **BMI>25**  **(n=66; 56%)** | |
| --- | --- | --- | --- | --- |
|  | **OR** | **95% CI** | **OR** | **95% CI** |
| **Clinical response** |  |  |  |  |
| Any csDMARD | 1.50 | 0.32-6.98 | 2.98 | 0.37-23.80 |
| MTX [±SSZ] | 1.65 | 0.30-9.08 | 4.52 | 0.36-56.78 |
| SSZ | 1.19 | 1.11-12.63 | 2.42 | 0.27-22.02 |
| **Remission** |  |  |  |  |
| Any csDMARD | 0.61 | 0.18-2.05 | 4.72 | 0.90-24.69 |
| MTX [±SSZ] | 0.70 | 0.18-2.70 | 5.73 | 0.74-44.50 |
| SSZ | 0.44 | 0.65-3.00 | 4.19 | 0.68-25.80 |

The adjusted multivariable logistic regression analysis included 119 patients. Two different models are presented for the following outcomes: clinical response and remission. Odds ratio (OR) and 95% confidence interval (CI) were calculated. All models were adjusted for age, gender, disease duration, HLA-B27 and baseline ASDAS. BMI, body mass index; HLA-B27, human leucocyte antigen B27; ASDAS, Ankylosing Spondylitis Disease Activity Score; csDMARD, conventional synthetic disease-modifying anti-rheumatic drug; MTX, methotrexate; SSZ, sulfasalazine.

**
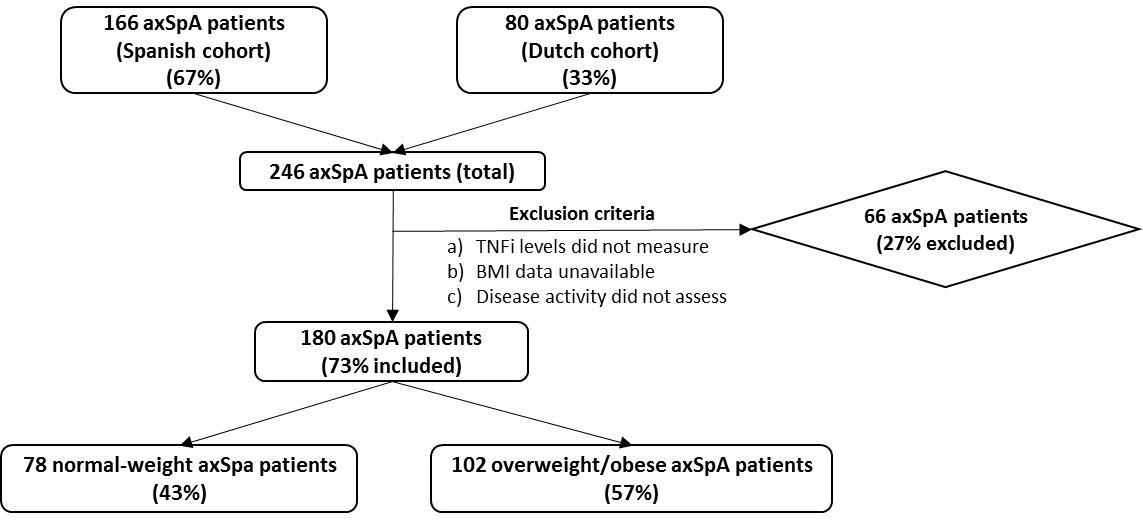
Flowchart of patients included in the present study**
